# Improvement of the Foaming Agent Feeding Process to an Electric Arc Furnace by Analyzing the Sound Generated by an Electric Arc and the Coefficient of Variation of Active Power Consumption

**DOI:** 10.3390/ma17235860

**Published:** 2024-11-29

**Authors:** Józef Schwietz, Bogdan Panic, Mariola Saternus, Jacek Pieprzyca, Krystian Janiszewski

**Affiliations:** 1 Stilmar Częstochowa, 42-202 Częstochowa, Poland; jozef.schwietz@stilmar.eu; 2 Faculty of Materials Engineering, Silesian University of Technology, 40-019 Katowice, Poland; bogdan.panic@polsl.pl (B.P.); jacek.pieprzyca@polsl.pl (J.P.); krystian.janiszewski@polsl.pl (K.J.)

**Keywords:** electric arc furnace (EAF), power consumption fluctuations, active power, noise emission, coefficient of variation

## Abstract

Electric arc furnaces are commonly used in foamed slag technology for the production of steel from steel scrap through an electric process. The effects of using this technology include increased efficiency, reduced consumption of refractory materials, reduced energy consumption, reduced electrode wear, and improved arc stability. The world is constantly looking for solutions to optimize the feeding of the foaming agent to the electric furnace, including determining the moment of starting its feeding. The authors propose using two parameters to determine the optimal moment of introducing the foaming agent: the change in the sound level emitted by the arc furnace and the fluctuations in active power consumption. In order to determine the above parameters, tests were carried out on an industrial alternating UHP arc furnace with a capacity of 70 tons. The sound intensity level was determined at which the feeding of the foaming agent to the furnace’s working space should begin. A moving coefficient of power consumption variation was developed and decision variables of the software for online foaming agent feeding were determined. As a result of implementing the developed solutions to the electric furnace control system and conducting comparative tests, savings were obtained in the form of reduced foaming agent consumption.

## 1. Introduction

The continuous development of iron-carbon alloy production technology has made steel the basic construction material, despite the intensive development of plastics. In order for steel to maintain its leading position, modern technologists are constantly looking for new methods of producing steel of increasingly better quality and with wider applications, lower environmental impact, and lower production costs [1,2,3,4,5].

Two methods of steel production dominate in the world. In integrated steelworks (with a full production cycle), pig iron is produced in blast furnaces and processed into steel using oxygen converters with the addition of steel scrap. The second method involves the production of steel from steel scrap through an electric process in steelworks equipped with arc furnaces. The steel production process in an electric arc furnace consists of four stages: furnace charging, scrap melting, converting the liquid metal (including slag foaming), and tapping. It is very important to note that the quality of scrap determines the steel yield from the melt [6].

Currently, all large steelworks in Europe that use arc furnaces for melting scrap use foamed slag technology. The effects of using this technology include increased productivity, reduced consumption of refractory materials, reduced energy consumption, reduced electrode wear, and improved arc stability [7,8,9]. This technology allows an increase in the efficiency of converting electrical energy into thermal energy in an electric arc furnace from about 55% for non-foamed slag to 85% for foamed slag [10]. Economical operation of arc devices is advantageous when using the highest possible voltages on the secondary side of the transformer and the lowest possible arc currents, i.e., when using so-called “long arcs”. This creates more favorable working conditions for expensive graphite electrodes, minimizing their wear, but at the same time, it worsens the working conditions of the refractory lining of the walls and ceiling due to the increased amount of radiated thermal energy. As a result, this leads to an increase in the consumption of refractory materials and an increase in the intensity of water cooling of water-cooled wall and ceiling elements, which contributes to an increase in associated heat losses.

An effective solution to the problem of the adverse effect of “long” electric arcs on the walls and ceiling of the furnace is the use of the slag foaming technique, thanks to which electric arcs burn the slag covering the metal bath along its entire length. Slag foaming should be started as early as possible, as it significantly reduces the fluctuations in active power consumption, which results in more uniform furnace operation and increases the efficiency of converting electrical energy into thermal energy in the electric arc furnace [11,12]. Currently, coke- or anthracite-based materials (foaming agents) are used for slag foaming—the main properties and chemical composition are presented in Table 1.

Slag foams under the influence of the formation of gas bubbles in its volume. The basic factor in the formation of the gas phase during slag foaming in metallurgical processes is carbon monoxide, which is formed in the volume of slag or metal-slag-gas emulsion, but the source of gas can also be water vapor from moisture and water from leaks in the cooling system [14]. In the process of melting steel in an arc furnace, carbon monoxide bubbles can be formed in the slag volume or transferred to the slag from the metal bath volume. Therefore, slag foaming occurs mainly due to the formation of carbon monoxide gas from the following reactions:

Cs + ½ {O_2_} = {CO}, (1)

(FeO) + {CO} = [Fe] + {CO_2_}, (2)

{CO_2_} + Cs = 2{CO}, (3)

[C] + (FeO) = [Fe] + {CO},(4)

Reaction (1) takes place during simultaneous injection of coal and oxygen by means of lances. Reactions (2) and (3) are partial reduction reactions of FeO contained in the slag by means of injected coal particles, where reaction (2) takes place at the slag–gas bubble boundary surrounding the solid coal particle and reaction (3) takes place at the interface between the surface of a solid carbon particle and the surrounding gas bubble. Gaseous CO is also formed in the arc furnace as a result of reaction (4)—reduction of FeO in slag from coal dissolved in a metal bath [8,9].

The most pressing problem facing the steel industry in the 21st century is climate change. The EAF process is very energy intensive and contributes to the increase in direct CO_2_ emissions in steel production. Therefore, extensive research is being conducted worldwide to reduce the electricity consumption and CO_2_ emissions of EAFs. Over the last decade, this research has focused on numerical and simulation modeling of individual furnace systems [15,16,17] and EAF process steps [18]; the use of biochar as a foaming agent [19]; the slag foaming process using plastic and rubber waste, carbonates, and nitrates and the slag foaming process with exogenous gas injection [20]; optimization of the conventional slag foaming process [21,22,23]; and the prediction of [C] content in molten steel obtained from scrap [24]. Reducing carbon dioxide emissions and lowering production costs require searching for solutions aimed at optimizing feeding of the foaming agent to the electric furnace, including determining the moment of starting its feeding. Unfortunately, there are few publications in this area, and the existing literature on determining the proper moment of starting feeding of the foaming agent to the electric furnace concerns primarily studies using acoustic signals (from an electric arc) and the vibrations of the furnace housing.

Nyss and Salmone [25] conducted tests on a 100-ton DC furnace. The foaming agent and oxygen were blown into the furnace by means of operating lances through the front window. An acoustic meter was installed on the wall of the furnace control room. The aim of the tests was to determine the relationship between the signals from the acoustic meter and the volume and quality of foamed slag present in the furnace, which would enable direct feeding of the foaming agent and oxygen. The acoustic signals of the sensor were recorded in a wide frequency band (from 0 to 5000 Hz) to determine the characteristic frequency ranges associated with slag foaming. A sharp decrease in the signal value was observed, especially in the frequency range of 100–150 Hz. It was confirmed that the presence of well-foamed slag in the longest part of the refining phase allowed for a reduction in electricity consumption in the range of 10 to 15 kWh/t, and a reduction in the nitrogen level in the metal bath from 10 to 20 ppm was also observed (for melts characterized by a similar metal charge).

Landa, Rodriguez et al. [26] conducted research aimed at finding the relationship between the sound emitted by the electric arc furnace (EAF), the harmonic distortions (THD) of the voltage and current arc waveform, and the quality of slag foaming [26]. The final effect of the research was the development of foaming agent and oxygen supply schemes, which allowed for shortening the power-on time and reducing the consumption of electrical energy. The research indicated the full usefulness of sound for the quantitative and qualitative description of slag foaming.

More advanced studies on the use of sound emitted by an arc furnace in two alternating current furnaces, each with a capacity of 100 t, were conducted by Dittmer, Kruger et al. [26] at Siemens AG. The studies used so-called structure-based sound, which is generated as a result of the spread of vibrations in a solid body. It should be emphasized that research on the use of structure-based sound was initiated in 1974 [27].

The research conducted at Siemens resulted in the development of the so-called FSM Manager (Foaming Slag Manager), which was used to identify the height of the foamed slag layer and to automatically control the supply of the foaming agent and oxygen to individual furnace zones [28]. Such control of the slag foaming process resulted in the stabilization of the electric arc and improved active energy consumption. Within a few weeks, the following parameters were reduced: power-on time, foaming agent consumption, and electrical energy consumption.

Further studies have shown the possibility of using structure-based sound to determine the amount of unmelted scrap, which is of great importance for process efficiency [29,30].

The Swedish Institute of Industrial Engineering and Management KTH, in its report, [31] emphasized the possibilities of using sound in the process of controlling slag foaming but pointed out the need to find an appropriate sound frequency that would help to describe the processes taking place in the furnace. The location of microphone installation was considered important, indicating that it should be located opposite the slag window or in a place where the sound signal could be recorded by the microphone without any obstacles. The possibility of using the harmonic distortion factor (THD) to analyze the course of slag foaming in the furnace was also considered. The unit of THD is a percentage. In the project mentioned in the report, 43 melts were analyzed and it was found that, at a THD value of 3%, the electric arc was completely covered with slag. This was also confirmed by the measurement of the sound level, which dropped significantly after reaching 3 percent THD.

Erives-Sanchez and Micheloud-Vernact [32] conducted a study to find indicators that informed about the degree of arc coverage by foamy slag. A strong relationship was found between furnace shell vibrations and the degree of electric arc coverage. The study used non-contact technology based on a laser vibrometer and an artificial neural network (ANN) to analyze the obtained results. It was also shown that the vibrations of the furnace shell were strongly correlated with the slag coverage of the electric arc. It was also proven that it was possible to safely measure the vibrations of an arc furnace by keeping sensitive electronic equipment away from the surrounding areas of the furnace, where most of the electronic devices attached to the furnace walls or near it failed.

The examples cited above indicate that the methods used so far to determine the optimal moment of introducing a slag foaming agent into an electric arc furnace are not very precise. Numerous works are being carried out in this direction, but the literature lacks information on the industrial and long-term use of such solutions. This is due to the fact that the operating conditions of an electric arc furnace are extremely difficult owing to the dynamic processes taking place within it, which depend on many factors. Precise identification of the mechanism of these processes using sensors installed on the furnace is practically impossible. In this article, an attempt was made to solve the problem indicated above by proposing the use of two parameters to determine the optimal moment of introducing the foaming agent: the change in the sound level emitted by the electric arc furnace and the fluctuations in active power consumption.

## 2. Materials and Methods

**Figure 1:**
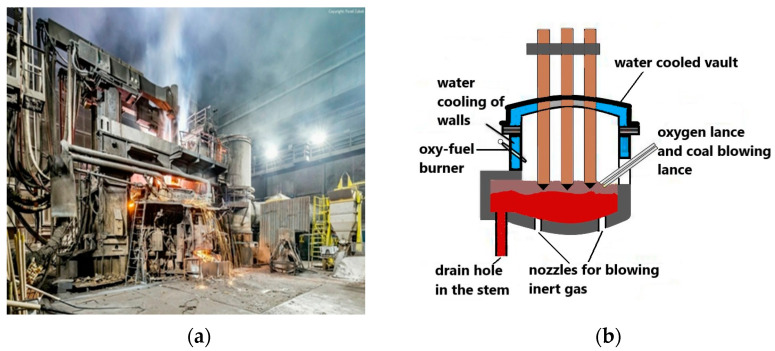
(**a**) Electric arc furnace used for testing, (**b**) schematic diagram of a typical arc furnace with its main components marked, own study based on [33].

The tests were carried out on an industrial electric arc furnace of UHP alternating current type (Figure 1) with a capacity of 70 tons. The furnace was supplied with electricity from a transformer with a capacity of 48 MVA.

In addition to electrical energy, chemical energy was supplied to the furnace (using three gas oxygen burners, each with a capacity of 3 MW). Two lances were used to feed the foaming agent, which was supplied from the main tank with a capacity of 30 M.

**Figure 2:**
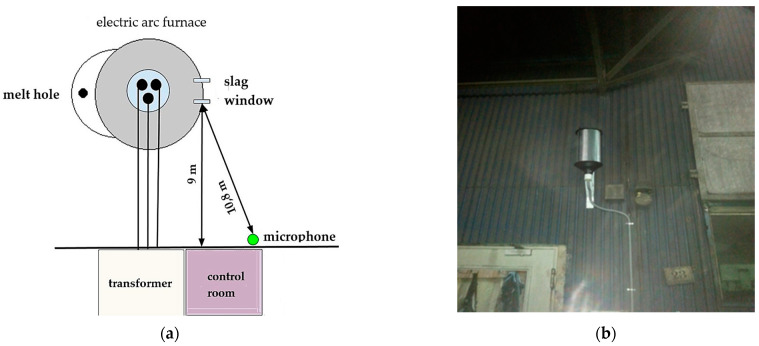
(**a**) The position of the microphone relative to the electric arc furnace, (**b**) the position of the microphone on the control room wall.

For the purpose of conducting the tests, a measuring system was designed, built, and configured, which was equipped with a sound level meter, a microphone, and a sound level meter controller with software for communication with the furnace control system. The position of the microphone in relation to the furnace and other objects located in the furnace hall is shown in Figure 2. This position guaranteed no thermal impact on the microphone.

**Figure 3:**
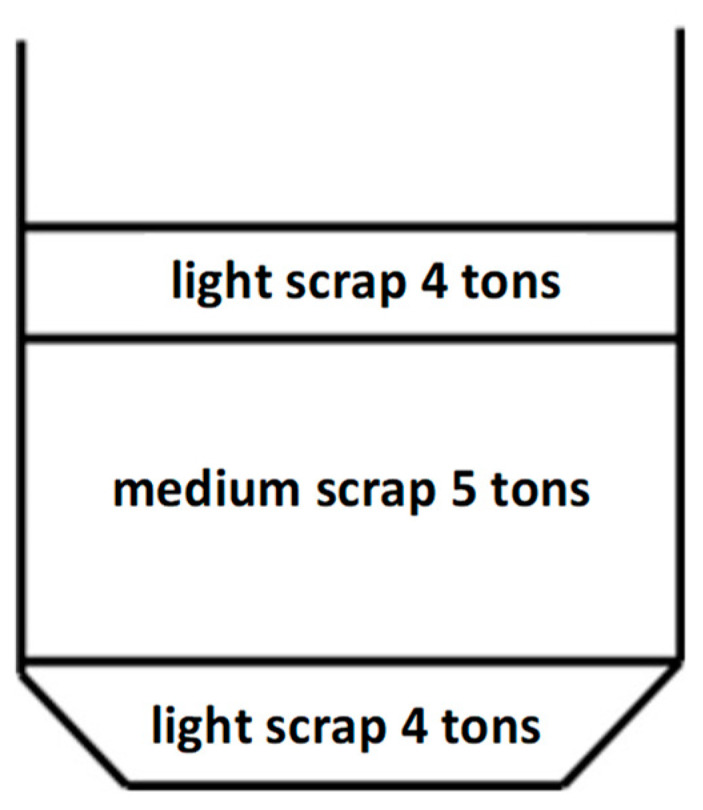
Distribution of scrap in the basket.

All research melts were conducted based on one scrap structure, which was as follows: light scrap—30 tons (scrap with thickness over 3 mm), medium scrap—26 tons (scrap with thickness over 6 mm), heavy scrap—16 tons (scrap with thickness over 10 mm), and scrap in the form of chips—2 tons. The scrap distribution in the basket is shown in Figure 3.

During the recording of the test melts, a foaming agent with the chemical composition shown in Table 2 was introduced into the furnace.

The research was divided into four stages:

I. Preliminary research conducted to determine the frequency of sound emitted by the working electric arc and research aimed at determining the sound level value at which the foaming agent should be supplied.

II. Research on active power consumption and the course of supplying the foaming agent to the furnace.

III. Research aimed at determining the value of the coefficient of variation of active power consumption (i.e., the moment of stabilization of active power consumption) below which the foaming agent should be supplied to the furnace.

IV. Verification of the obtained research results—conducting a series of 12 comparative melts.

The sound level meter was switched on after applying voltage to the furnace electrodes. The sound level emitted by the arc furnace was recorded every second in the third octave band at a frequency of 100 Hz. The furnace control system recorded the active power consumption and the course of feeding the foaming agent to the furnace. The measurement results were processed in a spreadsheet, which was used to generate graphs of the melting process. This allowed determining the sound level value at which the stabilization of power consumption began, even without feeding the foaming agent to the furnace’s working space. It was assumed that, at the moment of starting the stabilization of power consumption, the foaming agent should be fed to the furnace. The results of the preliminary studies are described in [34,35]. These studies indicated that one of the parameters determining the moment of feeding the foaming agent is the moment at which the sound level drops to 103 dB.

## 3. Results and Discussion

**Figure 4:**
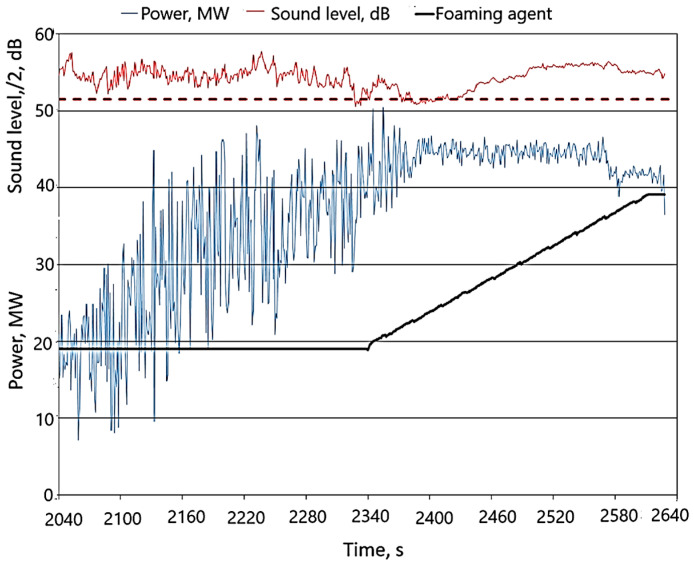
Active power consumption and sound level profiles recorded during the melting of one basket (black horizontal line (-) means that the foaming agent was not fed, a diagonal black line (/) means that this was the beginning of foaming agent feeding, (- - -) the dashed line refers to a sound level value of 103 dB).

The results of the study of active power consumption and the course of feeding the foaming to the furnace are shown in Figure 4 (for practical reasons, the noise level was divided by 2; in this situation, the reference noise level was, of course, 51.5 dB). The graph also schematically shows the process of feeding coal (foaming agent) to the furnace’s working space.

It was found that the sound level reached the reference value, but only for a dozen or so seconds. At that time, the foaming agent was added, although, as the discussed figure shows, the sound level increased a moment later by several dB. From that moment on, the sound level first dropped again to approx. 103 dB and then increased significantly until the end of the process, with the fluctuations in power consumption clearly stabilizing. Such observations were also made by analyzing graphs from other melts. These observations became the premise for taking into account the changes in power consumption fluctuations when choosing the optimal moment of feeding the foaming agent to the furnace. Figure 4 shows that, until the moment of clear stabilization, the power consumption increased and at the same time showed significant fluctuations. It was therefore decided to evaluate the average course of the increase in power consumption using a moving average, while the size of the fluctuations (dispersion) was evaluated using the standard deviation, of course, also treating it as a moving quantity. With this approach, in order to compare the results obtained for different alloys, it is advisable to use a relative measure of dispersion—the coefficient of variation, as follows:

(5) $$WZ=100\cdot \bar{x}/\hat{s}\left[\%\right],$$

where $\bar{x}$—arithmetic mean calculated based on an n-element sample:

(6) $$\bar{x}=\frac{\sum_{i}^{n}x_{i}}{n},$$

and $\hat{s}$—standard deviation estimator calculated from an n-element sample:

(7) $$\hat{s}=\sqrt{\frac{\sum_{i}^{n}{\left(x_{i}-\bar{x}\right)}^{2}}{\left(n-1\right)}}$$

The “movability” of the power consumption variation coefficient results from the fact that the sample elements (active power values) are shifted by one second in subsequent calculation steps. The values of the variation coefficient were calculated “backwards.” This means that they were assigned to the moment of the melt duration corresponding to the last second of the current n-second time interval.

In the first part of the statistical analysis, the width of the time interval for calculating the moving coefficient of variation of active power and the width of the time interval of its stable assumed value were determined. The nonparametric Friedman test was used, the application of which was dictated by the preliminary analysis of the normality of the distributions, to determine the time after which the foaming agent should be added. In the vast majority of cases, the verification of the normality of these distributions, carried out using the Shapiro–Wilk test [36], gave a negative result. In the discussed analysis, a post-hoc test was used in the variant proposed by Dunn, taking into account the so-called Bonferroni correction [37].

**Figure 5:**
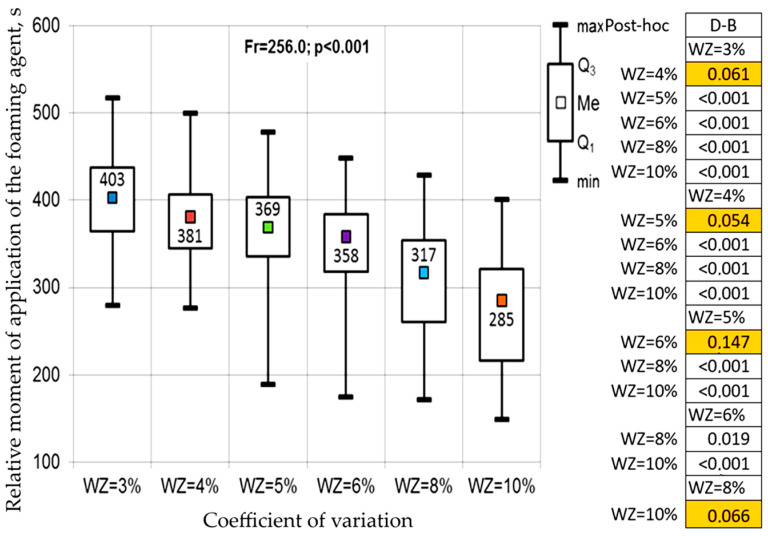
Results of the Friedman test and the Dunn–Bonferroni post-hoc test in assessing the significance of differences in the relative moment of foaming agent application in relation to the threshold values of the coefficient of variation of active power.

Based on the analysis, the values of two decision variables were assumed (the interval for calculating the coefficient of variation equal to 10 s and the stabilization time interval equal to 10 s) and the second part of the statistical analysis was performed. The Friedman test and the Dunn–Bonferroni post-hoc test were used again, but this time the analysis concerned the effect of the threshold level of the coefficient of variation on the moment of adding the foaming agent. The results of this analysis are presented in Figure 5. The figure uses the values of the median (Me) and quartiles 1 (Q1) and 3 (Q3).

It should be stated that the results of the Friedman test indicated (in general) statistically significant differences in the moments of foaming agent application depending on the adopted value of the coefficient of variation. Detailed conclusions were provided by the results of the post-hoc test. In both cases, there were no statistically significant differences between the moments of foaming agent application for neighboring values of the coefficient of variation. The only exception to this rule was the difference between series for coefficients of variation of 6% and 8%, which showed features of statistical significance. This effect was most likely caused by the first two percent jump in the threshold value of the coefficient of variation.

In the case of the five percent coefficient of variation, most arguments supported the choice of a ten-second interval for calculating its value.

Finally, the following procedure for controlling the foaming agent dosing unit could be formulated: the width of the interval for calculating the coefficient of variation of the motion is 10 s, the width of the stabilization interval of its value is 10 s, and the threshold value of the coefficient of variation is 5%.

**Figure 6:**
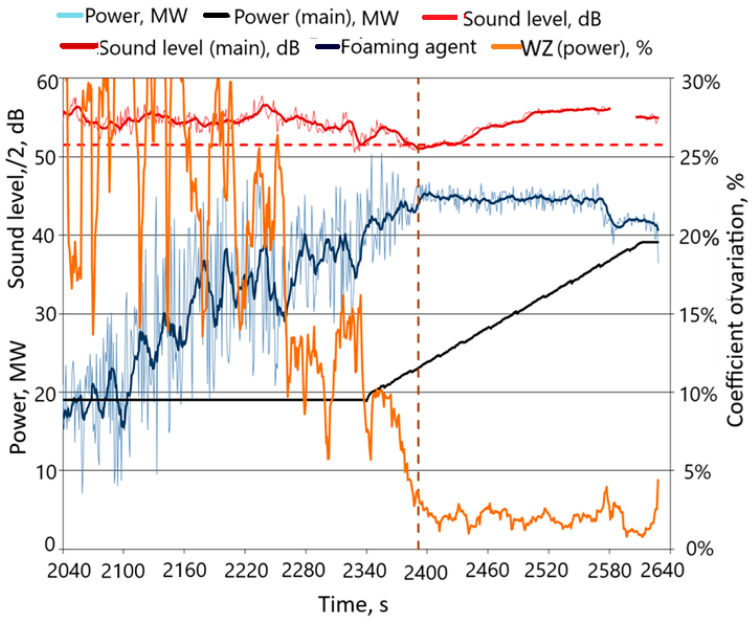
Active power consumption and sound level profiles recorded during the melting of one basket and graphs of moving averages and moving coefficients of variation supplemented with the moment of foaming agent application (black horizontal line (**-**) means that the foaming agent was not fed, a diagonal black line (**/**) means that this was the beginning of foaming agent feeding, (- - -) the dashed line refers to a sound level value of 103 dB).

After applying the procedure described here, the foaming agent application times reached the values shown in Figure 6.

### 3.1. Comparative Melt Results

In order to verify the obtained results, a series of 12 comparative melts (industrial tests) were carried out with and without the use of the designated parameters that determined the start of slag foaming agent feeding, i.e.,:

- Frequency of sound measured, 100 Hz;
- Sound level below which feeding of the foaming agent starts, 103 dB;
- Width of the interval for calculating the moving coefficient of variation, 10 s;
- Width of the stabilization interval of its value, 10 s;
- Threshold value of the coefficient of variation, 5%.

In order to conduct industrial research using the above parameters, an algorithm was developed containing a procedure for obtaining data and performing calculations to determine the moment of starting to feed coal to the furnace. Then, a program was developed that was implemented in the controller to control the feeding of the foaming agent to the furnace.

**Figure 7:**
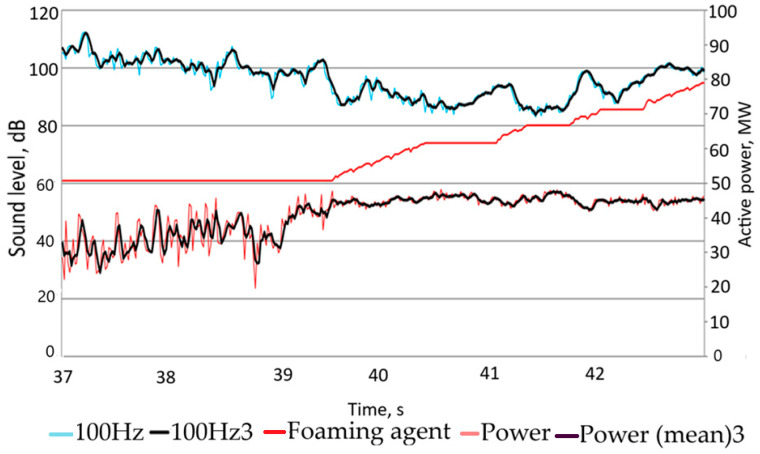
Starting of feeding of the foaming agent to the arc furnace under the control of the new program.

During all of these tests (using the designated parameters) a strong stabilization of power consumption was observed just a few seconds after starting to feed the foaming agent, at a low sound level (below 100 dB). The test results obtained during one of such tests are presented in Figure 7.

**Figure 8:**
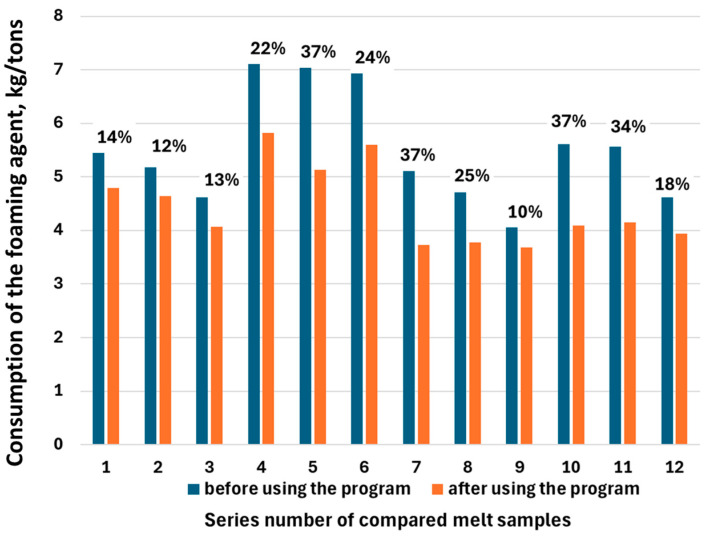
Comparison of foaming agent consumption during melts running with the new program determining the moment of starting foaming agent feeding and melts running with the old program. The percentage values indicate the difference in the value of foaming agent consumption before and after using the new program.

Then, in order to assess the effectiveness of the new program, which initiated feeding of the foaming agent to the furnace, the foaming agent consumption (kg/t of liquid steel) was compared during 12 melts working with the old program. The results of the comparison of these tests (melts) are presented in Figure 8. The figure shows the percentage value indicating the difference in the value of foaming agent consumption before and after using the new program. It was found that the use of the new method for determining the moment of starting the feeding of the foaming agent to the furnace resulted in a reduction of foaming agent consumption from 10% to even 37%.

### 3.2. Uniqueness and Novelty of the Approach

A literature review indicated that currently there is no industrially tested solution available that used the change in the sound level emitted by the arc furnace and the fluctuations in active power consumption to determine the moment of introducing the slag foaming agent. In several cases alternative solutions have been used, such as using acoustic signals from a sensor installed on the wall of the furnace control room [14], the sound emitted by the arc furnace (EAF) and distortions of the voltage and current waveform of the arc [25,30], structure-based sound to determine the amount of unmelted scrap [26,28,29], and vibrations of the furnace shell [31]. According to the study conducted by Dittmer, Kruger et al. [27], a reduction in foaming agent consumption by about 12% was achieved at Siemens AG. It should be noted that the results of the studies described herein achieved an average reduction in foaming consumption by about 24%. Of course there is still room for further research using different tools and methods aimed at solving the problem described in the article.

The novelty of the approach results from the fact that few scientific centers in the world have been able to find a solution that successfully ends with automatic feeding of the foaming agent to the EAF. The authors of the article managed to successfully develop an algorithm for initiating the introduction of the foaming agent to the EAF and implement this solution under industrial conditions. This is an innovative approach to the problem of automatic starting of feeding of the foaming agent that is simultaneously independent of human decision and the type of charge. This method is more universal than the solutions used so far. The approach proposed by the authors can be used for other EAFs but, due to the specificity of individual plants, common use of this solution is possible after appropriate calibration of the entire measurement system. The limitation is the distance of the microphone installation and if more than one furnace is operating in one hall. Then interference from the second furnace occurs. The sound level decreases with the square of the distance, but then the system loses sensitivity and resistance to sound interference. Interference can be filtered out, but in the case of one furnace, it is negligible.

## 4. Conclusions

The paper presents the results of a study on the optimization of the slag foaming agent feeding process to the EAF furnace. It was shown that proper problem definition can lead to a significant reduction in foaming agent consumption. Based on the conducted research, the following conclusions were drawn:

1. It was found that fluctuations in power consumption and the sound emitted by the furnace can be used to determine the optimal moment to start feeding the slag foaming agent to the electric arc furnace.
2. It was found that the sound intensity level at which the foaming agent should be fed into the furnace’s working space—under the analyzed research conditions—should be 103 dB at a frequency of 100 Hz.
3. As a result of the statistical analysis of fluctuations in the furnace’s active power consumption, a sliding coefficient of variation of power consumption was developed and decision variables for the on-line foaming agent feeding process control software were determined, as follows:
  – Width of the interval for calculating the sliding coefficient of variation—10 s;
  – Width of the stabilization interval of its value—10 s;
  – Threshold value of the coefficient of variation—5%.
4. As a result of implementing the developed solutions into the electric furnace control system and conducting research tests (melts), savings were found in the form of reduced foaming agent consumption, which is of significant importance for reducing the carbon footprint.

## Figures and Tables

**Table 1:** Properties of foaming materials produced on the basis of anthracite and coke [13].

Property	Anthracite-Based Materials	Coke-Based Materials
Carbon content	min. 90 wt.%	min. 85 wt.%
Sulfur content	max. 0.9 wt.%	max. 0.7 wt.%
Ash content *	max. 7 wt.%	max. 12 wt.%
Volatile matter content	max. 3 wt.%	max. 1.5 wt.%
Moisture content	max. 2.5 wt.%	max. 1.5 wt.%
Grain size	0 ÷ 3 mm	0 ÷ 3 mm

*—determined after burning the sample.

**Table 2:** Chemical composition of the foaming agent after burning.

Feature	Unit, wt.%
Moisture	2.8–4.4
Ash	4.2–12.4
Volatile parts	1.6–5.8
Carbon	72.11–88.77
Sulfur	0.77–1.03

## Data Availability

Data available upon request.
